# Anoikis resistance and immune escape mediated by Epstein-Barr virus-encoded latent membrane protein 1-induced stabilization of PGC-1α promotes invasion and metastasis of nasopharyngeal carcinoma

**DOI:** 10.1186/s13046-023-02835-6

**Published:** 2023-10-07

**Authors:** Chaoliang Liao, Min Li, Xue Chen, Chenpeng Tang, Jing Quan, Ann M. Bode, Ya Cao, Xiangjian Luo

**Affiliations:** 1 NHC Key Laboratory of Carcinogenesis and Hunan Key Laboratory of Oncotarget Gene, Hunan Cancer Hospital and The Affiliated Cancer Hospital of Xiangya School of Medicine, Central South University, Changsha, Hunan 410013 PR China; 2 Key Laboratory of Carcinogenesis and Invasion, Chinese Ministry of Education, Cancer Research Institute, School of Basic Medicine, Central South University, Changsha, Hunan 410078 PR China; 3 Department of Medical Science Laboratory, The Fourth Affiliated Hospital of Guangxi Medical University, Liuzhou, Guangxi 545007 PR China; 4 Department of Oncology, Nanjing Hospital of Chinese Medicine Affiliated to Nanjing University of Chinese Medicine, Nanjing, Jiangsu 210023 PR China; 5 Early Clinical Trial Center, Hunan Cancer Hospital and The Affiliated Cancer Hospital of Xiangya School of Medicine, Central South University, Changsha, Hunan 410013 PR China; 6 The Hormel Institute, University of Minnesota, Austin, MN 55912 USA; 7 National Health Commission (NHC) Key Laboratory of Nanobiological Technology, Xiangya Hospital, Central South University, Changsha, Hunan 410078 PR China

**Keywords:** LMP1, PGC-1α, Anoikis resistance, Immune escape, Nasopharyngeal carcinoma

## Abstract

**Background:**

Epstein-Barr virus (EBV) is the first discovered human tumor virus that is associated with a variety of malignancies of both lymphoid and epithelial origin including nasopharyngeal carcinoma (NPC). The EBV-encoded latent membrane protein 1 (LMP1) has been well-defined as a potent oncogenic protein, which is intimately correlated with NPC pathogenesis. Anoikis is considered to be a physiological barrier to metastasis, and avoiding anoikis is a major hallmark of metastasis. However, the role of LMP1 in anoikis-resistance and metastasis of NPC has not been fully identified.

**Methods:**

Trypan blue staining, colony formation assay, flow cytometry, and TUNEL staining, as well as the detection of apoptosis and anoikis resistance‐related markers was applied to evaluate the anoikis-resistant capability of NPC cells cultured in ultra-low adhesion condition. Co-immunoprecipitation (Co-IP) experiment was performed to determine the interaction among LMP1, PRMT1 and PGC-1α. Ex vivo ubiquitination assay was used to detect the ubiquitination level of PGC-1α. Anoikis- resistant LMP1-positive NPC cell lines were established and applied for the xenograft and metastatic animal experiments.

**Results:**

Our current findings reveal the role of LMP1-stabilized peroxisome proliferator activated receptor coactivator-1a (PGC-1α) in anoikis resistance and immune escape to support the invasion and metastasis of NPC. Mechanistically, LMP1 enhances PGC-1α protein stability by promoting the interaction between arginine methyltransferase 1 (PRMT1) and PGC-1α to elevate the methylation modification of PGC-1α, thus endowing NPC cells with anoikis-resistance. Meanwhile, PGC-1α mediates the immune escape induced by LMP1 by coactivating with STAT3 to transcriptionally up-regulate PD-L1 expression.

**Conclusion:**

Our work provides insights into how virus-encoded proteins recruit and interact with host regulatory elements to facilitate the malignant progression of NPC. Therefore, targeting PGC-1α or PRMT1-PGC-1α interaction might be exploited for therapeutic gain for EBV-associated malignancies.

**Supplementary Information:**

The online version contains supplementary material available at 10.1186/s13046-023-02835-6.

## Background

Epstein-Barr virus (EBV) is the first discovered human tumor virus that is associated with a variety of malignancies of both lymphoid and epithelial origin, such as Burkitt lymphoma, Hodgkin lymphoma, gastric carcinoma (GC), and nasopharyngeal carcinoma (NPC) [1–3]. The EBV-encoded latent membrane protein 1 (LMP1) has been well-defined as a potent oncogenic protein, which is intimately correlated with NPC pathogenesis [4–8]. High frequencies of *LMP1* mRNA expression have been detected in NPC biopsies (> 80%) and in nasopharyngeal swabs from NPC patients (> 90%) [5]. LMP1 belongs to the tumor necrosis factor receptor superfamily and is an integral membrane protein. It consists of a short cytoplasmic N-terminus, six transmembrane domains (TMs), and a large cytoplasmic C-terminal domain. The C-terminus represents the main functional region of LMP1 to mediate signal transduction. Three distinct subdomains have been identified as C-terminal activating regions 1, 2, and 3 (CTAR1,2,3) [9–11]. LMP1 can induce the transformation of epithelial cells, and promote cellular metabolism, proliferation, invasion, and metastasis by directly activating a variety of signaling cascades. These include NF-κB, MAPKs, PI3-K/AKT, and JAK/STAT pathways; and the TMs and CTAR regions of LMP1 mainly mediate these processes [4, 8, 9, 12–14]. In addition, LMP1 has been reported to interact with receptor interacting protein kinases (RIPKs) through its CTAR2 domain, thus protecting NPC cells from necroptotic cell death [7].

Anoikis is a particular type of apoptosis triggered by loss of cell–matrix interactions. During the process of metastasis, cancer cells detach from the extracellular matrix and infiltrate into the circulatory system, and only a few of them acquire resistance to anoikis, eventually forming secondary tumors in distant organs [15, 16]. Accumulating evidence has shown that resistance to anoikis is a critical characteristic of highly aggressive cancer cells, and represents one of the malignant phenotypes that boost cancer metastasis [17, 18]. Studies have shown that EBV-infected cells have the intrinsic ability to resist anoikis [19], and LMP1-meditated signaling is involved in anoikis resistance [20–23]. However, the underlying mechanism has not been fully clarified.

Peroxisome proliferator activated receptor (PPAR) coactivator-1a (PGC-1α) is the most well-studied member of the PGC-1 family coactivators, and is extensively involved in the regulation of cell metabolism, apoptosis, invasion, and radiochemoresistance of cancer cells [24–28]. PGC-1α expression has been shown to be required for anchorage-independent growth of mouse embryo fibroblasts (MEFs) [28]. Clinical analysis of invasive breast cancer manifested a significant correlation between high PGC-1α expression in invasive cancer cells and distant dissemination. Moreover, over 80% of circulating cancer cells (CTC) harvested from patients with metastatic breast cancer were observed to be PGC-1α-positive [26]. In addition, our previous studies have demonstrated that PGC-1α is intimately involved in TGFβ1-induced epithelial-mesenchymal transition (EMT) and radiotherapy resistance of NPC cells [24, 25].

As a typical coactivator, PGC-1α interacts with a variety of nuclear receptors to initiate the transcriptional program of target genes. It is a short-lived protein, and its stability and activity are subject to multiple post-translational modifications [29–41], among which arginine methylation modification has an important role. Arginine methylation is involved in the regulation of a variety of cellular processes, including protein subcellular localization [42], protein- protein interactions [43], transcriptional activation [39, 44], and protein stability [45, 46]. PRMT1 is a major member of the arginine methyltransferase (PRMT) family in mammalian cells [47, 48]. PGC-1α can be methylated by PRMT1, which potentiates PGC-1α coactivator activity [39].

Immune escape is critical for the survival and malignant progression of cancer cells [49–52]. The programmed cell death protein 1 (PD-1) /programmed cell death ligand 1 (PD-L1) immune checkpoint pathway plays a pivotal role in the regulation of T cell exhaustion and dysfunction [51]. This immune checkpoint prevents tumor antigen-specific T cells from being effectively activated, thus promoting tumor immune escape [53]. In the process of metastasis, anoikis-resistant cells must escape from the recognition and destruction of the immune system, facilitating their survival and distant dissemination.

In the present study, we explored the role of LMP1-stabilized PGC-1α in anoikis resistance and immune escape to support the invasion and metastasis of NPC. Mechanistically, LMP1 enhances PGC-1α protein stability by promoting the PRMT1-PGC-1α interaction to elevate the arginine methylation of PGC-1α, thus enabling NPC cells to be resistant to anoikis. Meanwhile, PGC-1α mediates the immune escape induced by LMP1 by coactivating with STAT3 to transcriptionally up-regulate PD-L1 expression. The present study aims to clarify the intimate interactions between EBV-encoded proteins and host cells, which facilitates the anoikis resistance and aggressive phenotype of NPC. These actions further provide a novel perspective for developing therapeutics against EBV infection-related tumors.

## Materials and methods

### Cell culture

CNE1 and HNE2 are LMP1-negative NPC cell lines. CNE1-LMP1 (CM) is an LMP1-transfected CNE1 cell line and HNE2-LMP1 (HM) is an LMP1-transfected HNE2 cell line. Cells were grown in RPMI-1640 media (Gibco BRL) supplemented with 10% v/v heat-inactivated fetal bovine serum (FBS), 1% w/v penicillin, and 1% w/v streptomycin, and cultured at 37°C in a humidified incubator containing 5% CO_2_.

### Reagents and antibodies

The LMP1 antibody was purchased from DAKO (Cambridge, MA, USA) and the β-actin antibody was from Sigma-Aldrich (St. Louis, MO, USA). The cleaved-PARP and cleaved-Caspase 3 antibodies were obtained from Cell Signaling Technology (Danvers, MA, USA). Ubiquitin and PD-L1 antibodies were purchased from Proteintech (Chicago, IL, USA). The Flag-Tag monoclonal antibody and Myc-Tag monoclonal antibody antibodies were from Immunoway Biotechnology Company (Plano, Texas, USA). The PGC-1α antibody was obtained from Novus Biologicals (Littleton, CO, USA) and the PRMT1 antibody was purchased from Santa Cruz Biotechnology (Santa Cruz, CA, USA). The dimethyl arginine antibody was obtained from ImmuneChem Pharmaceuticals Inc (Burnaby British Columbia, Canada). Cycloheximide was obtained from Sigma-Aldrich (Saint Louis, MO, USA), and MG132 was purchased from MedChemExpress (Shanghai, China).

### Anoikis resistant cell survival rate assay

The same number of cells was seeded and suspended in ultra-low attachment 6-well plates (3471, Corning) for 0, 2, 4 or 6 days. The cells were then stained with 0.4% trypan blue (T10282, Invitrogen) and counted.

### TUNEL assay

Apoptosis was detected using the YF488 TUNEL Kit (UELandy, Suzhou, China), and the specific operation process was performed according to the manufacturer’s suggested protocol. First, cells were collected and washed twice with PBS. Cells were then fixed with 4% paraformaldehyde for 30 min, and then 0.2% Triton X-100 was added to permeate the cells. Cells were treated with TUNEL reaction mixture at 37℃ for 60 min in dark conditions. After centrifuging and discarding the supernatant fraction, cells were suspended with 0.1% Triton X-100 (containing 5 mg/ml BSA) and washed twice. The number of cells was determined by DAPI staining, and the samples were observed under a fluorescence microscope (Agilent, Palo Alto, USA).

### Ex vivo ubiquitination assay

For the PGC-1α ubiquitination assay ex vivo, 293T cells were transfected with HA-Ub, Flag-PGC-1α-WT or Flag-PGC-1α-Mut and PRMT1 or shPRMT1 for 48 h, and then treated with 20 μM MG132 for 10 h. The cells were disrupted for 40 min in IP lysate buffer containing a protease inhibitor. The cell lysates were then heated at 95°C for 10 min and diluted tenfold in a non-SDS buffer, and the samples were rotated and incubated at 4°C for 30 min. The lysis mixture was then centrifuged to obtain the cytoplasmic protein portion. Flag-PGC-1α was immunoprecipitated with anti-Flag at 4°C overnight, and then incubated with Dynabeads®Protein A/G (B23202, Bimake). The samples were washed and used for immunoblot analysis.

### Co-culture experiments and flow cytometry

Co-cultures at a 5:1 (T cells or Jurkat: silencing or overexpressing PGC-1α in NPC cells) ratio were incubated for 48 h and the Annexin V PE/7-AAD apoptosis detection Kit (FXP027, 4A Biotech) was used to measure apoptosis.

### Enzyme-linked immunosorbent assay for detection of IFN-γ

The transfected tumor cells were co-cultured with activated T cells for 24 h or with Jurkat cells for 24 h. The culture supernatant fraction was collected to detect IFN-γ. All experiments were performed according to the manufacturer's instructions for the IFN-γ ELISA Kit (CHE0017, 4A Biotech).

### Animal studies

Animal procedures were in accordance with the standards established by the guidelines for the Care and Use of Laboratory Animals of Central South University (Changsha, China). For the xenograft tumor models, female Balb/C nude mice (*n* = 4) aged 4 to 6 weeks were subcutaneously injected with approximately 2 × 10^7^ CM-AR-con and AR-shPGC-1α cells (suspended in 100 µL 1640), respectively, on the left and right sides. The tumor size was measured with a caliper every 3 days and the tumor volume was calculated: volume = (L × W^2^/2). After 5 weeks, the tumor was removed and weighed, and the tissue was embedded in paraffin or frozen with liquid nitrogen for further examination.

For the metastatic tumor model, CM-AR-con or AR-shPGC-1α cell suspensions (2 × 10^6^ cells suspended in 100 μl 1640 for each mouse) were injected into the lateral tail vein of 4-week-old BALB/c nu/nu mice. After 8 weeks, the mice were euthanized by inhalation of CO_2_. Lung tissues were collected and the number of pulmonary metastatic nodules was counted. The research protocol was approved by the Ethics Committee of Xiangya School of Medicine, Central South University.

## Results

### LMP1 promotes anoikis resistance and invasion of NPC cells

**Fig. 1:**
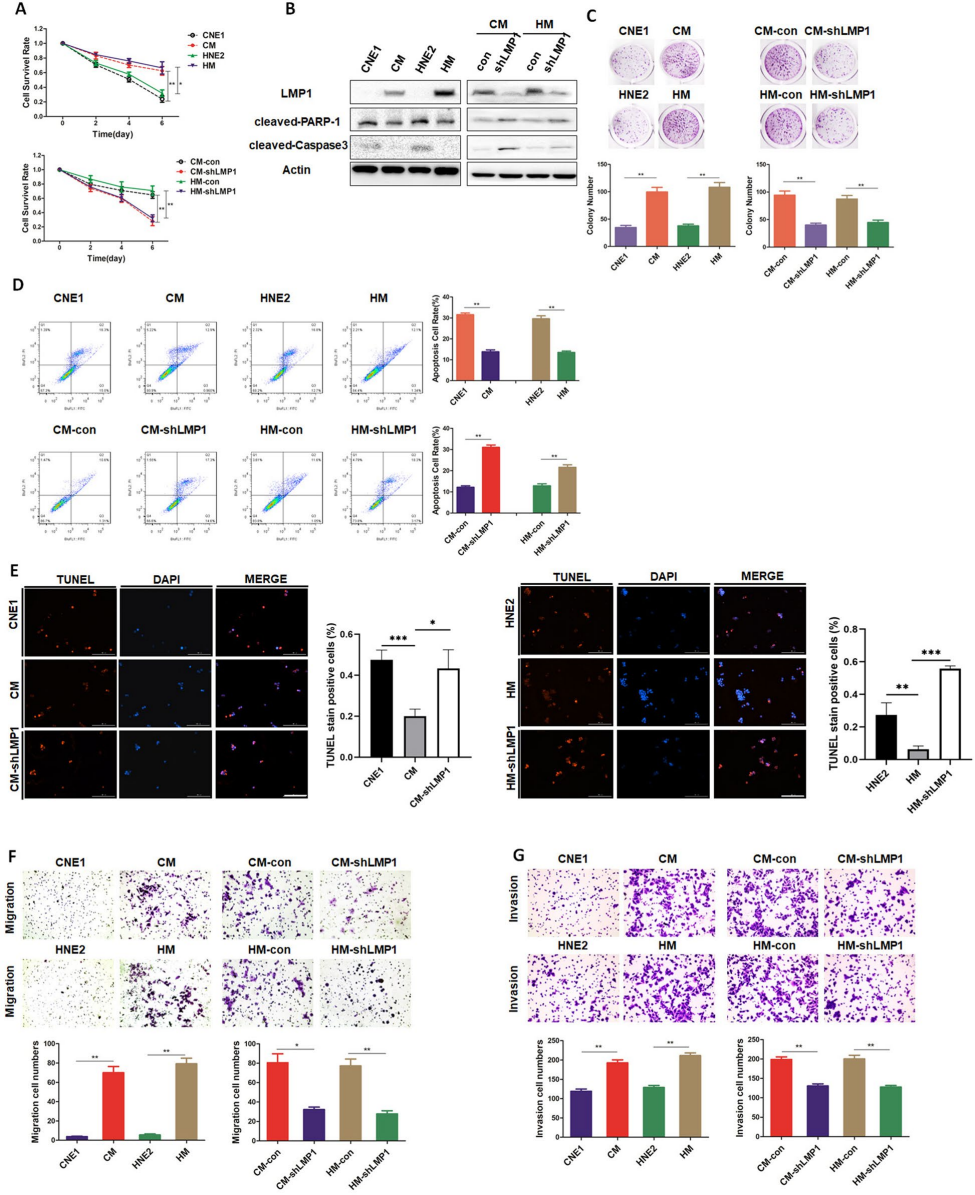
LMP1 promotes anoikis resistance and invasion of NPC cells. **A** Cell survival rate of CNE1/CM, HNE2/HM, CM-con/CM-shLMP1, HM-con/HM-shLMP1 cells after 0, 2, 4, or 6 days suspension (**p < 0.01, *p < 0.05). **B** CNE1/CM, HNE2/HM, CM-con/CM-shLMP1, HM-con/HM-shLMP1 cells were cultured in suspension for 48 h, and the expression of LMP1, cleaved-PARP-1, and cleaved-Caspase 3 was detected by Western blotting. **C** CNE1/CM, HNE2/HM, CM-con/CM-shLMP1, and HM-con/HM-shLMP1 cells were cultured in suspension for 7 days, and the colony formation rate of the cells reattached was observed (**p < 0.01, *p < 0.05). **D** CNE1/CM, HNE2/HM, CM-con/CM-shLMP1, and HM-con/HM-shLMP1 cells were cultured in suspension for 48 h, and the apoptotic rate of cells was detected by flow cytometry (**p < 0.01). **E** CNE1/ CM/CM-shLMP1 and HNE2/HM/HM-shLMP1 cells were cultured in suspension for 48 h, and the apoptotic rate of cells was detected by TUNEL assay, respectively (***p < 0.001,**p < 0.01, *p < 0.05). bar, 200μm. CNE1/CM, HNE2/HM, CM-con/CM-shLMP1, and HM-con/HM-shLMP1 cells were cultured in suspension for 7 days, and (**F**) the migration and (**G**) invasion abilities were detected by cell migration assay (**p < 0.01, *p < 0.05)

The LMP1-negative NPC cell lines CNE1 and HNE2 and the corresponding LMP1-positive NPC cell lines CNE1-LMP1 (CM) and HNE2-LMP1 (HM) are all attached cells. These two groups of NPC cell lines were cultured in low-adhesion petri dishes to mimic the condition of loss of adhesion. We counted the number of viable cells by using trypan blue staining or detected the viability of cells by the 3-(4,5- dimethylthiazol-2-yl)- 5-(3-carboxymethoxyphenyl)-2-(4- sulfophenyl)-2H- tetrazolium (MTS) assay. We observed that LMP1-positive NPC cells had a higher survival rate than the corresponding LMP1-negative cells (Fig. 1A, Supplementary Fig. 1A). Moreover, knocking down LMP1 substantially decreased the survival rate in NPC cells (Fig. 1A, Supplementary Fig. 1). Lower expression of typical markers of apoptosis, cleaved-PARP-1 and cleaved-Caspase 3, whereas higher expression of anoikis resistance-related protein Bcl-2 was found in LMP1-positive cells in suspension culture compared to those in LMP1-negative cells (Fig. 1B, Supplementary Fig. 2A). Knockdown of LMP1 rescued the expression of apoptotic markers, while suppressing anoikis resistance-related protein expression (Fig. 1B, Supplementary Fig. 2A), and similar results were observed when knocking down LMP1 in EBV-positive NPC cell line C666-1 (Supplementary Fig. 2B). In addition, the results of colony formation assays also agree with these observations (Fig. 1C). CNE1, CM, HNE2, and HM cells were cultured in suspension for 48 h, and apoptosis was measured by flow cytometry. The results showed that the apoptotic rate of LMP1-positive cells was markedly lower than that of LMP1-negative cells, whereas silencing LMP1 enhanced apoptosis (Fig. 1D). TUNEL staining further confirmed this observation (Fig. 1E). Moreover, cells were cultured in suspension for 7 days, and then cell migration and invasion experiments were performed. The results demonstrated that the number of migrated and invasive cells was significantly increased in the LMP1-positive group, and these phenomena were reversed by knockdown of LMP1 (Fig. 1F-G). Thus, these findings support that LMP1 enhances anoikis-resistance, migration, and invasion of NPC cells in suspension culture.

### PGC-1α mediates the anoikis resistance induced by LMP1 in NPC cells

**Fig. 2:**
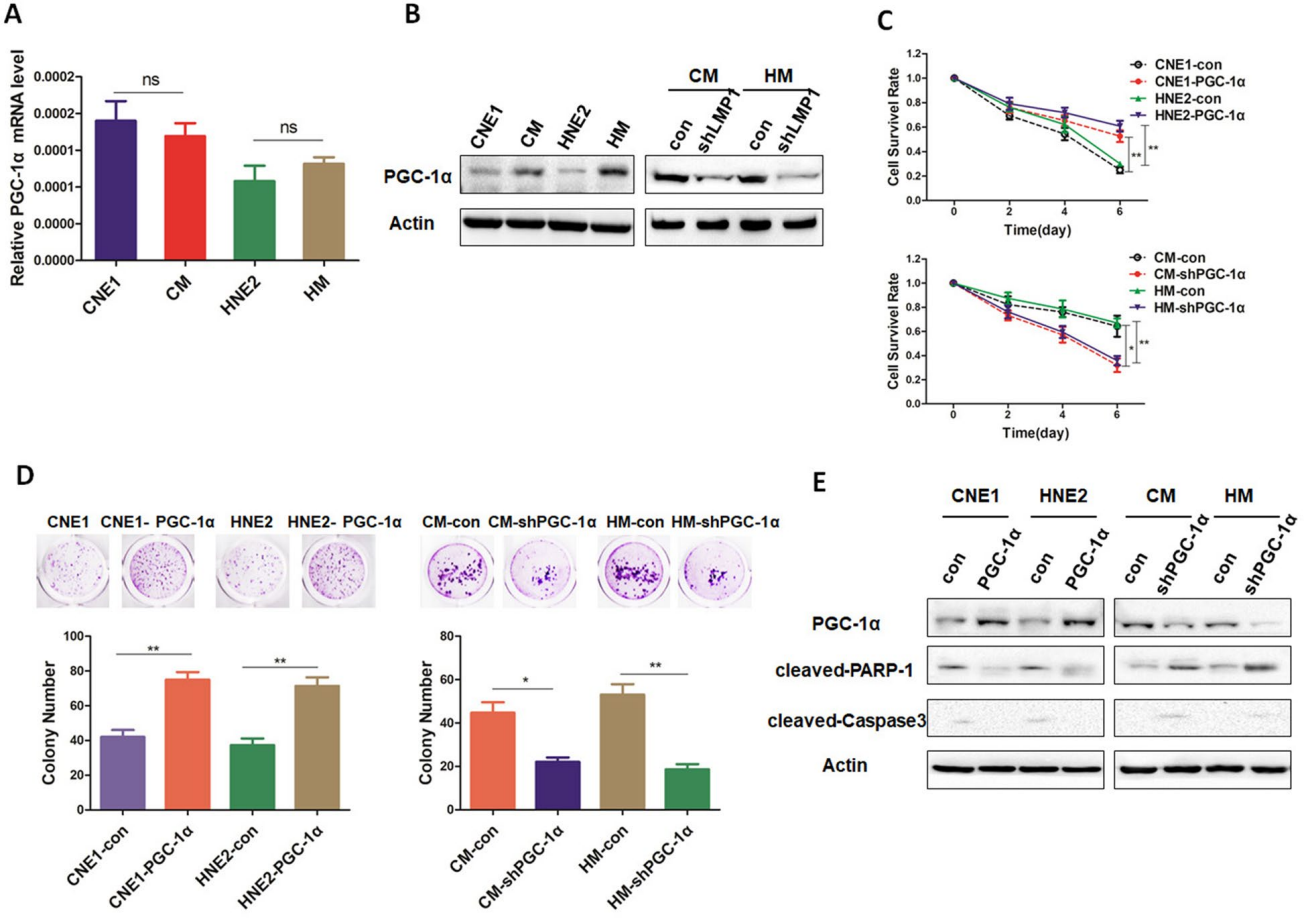
PGC-1α mediates LMP1-induced anoikis resistance in NPC cells. **A** CNE1/CM and HNE2/HM cells were cultured in suspension for 48 h, and then the transcription level of PGC-1α was detected by real-time fluorescence quantitative PCR. **B** CNE1/CM, HNE2/HM, CM-con/CM-shLMP1, and HM-con/HM-shLMP1 cells were cultured in suspension for 48 h, and the protein levels of PGC-1α were detected by Western blotting. **C** Survival rate of CNE1-con/CNE1-PGC-1α, HNE2-con/HNE2-PGC-1α, CM-con/CM-shPGC-1α, and HM-con/HM- shPGC-1α cells was assessed after 0, 2, 4, and 6 days suspension culture (**p < 0.01, *p < 0.05). **D** CNE1-con/CNE1-PGC-1α, HNE2-con/HNE2-PGC-1α, CM-con/CM-shPGC-1α, and HM-con/HM- shPGC-1α cells were cultured in suspension for 7 days, and the colony formation rate of the cells reattached was measured (**p < 0.01, *p < 0.05). **E** CNE1-con/CNE1-PGC-1α, HNE2-con/HNE2- PGC-1α, CM-con/CM-shPGC-1α, and HM-con/HM-shPGC-1α cells were cultured in suspension for 48 h and Western blotting was used to detect the expression of PGC-1α, cleaved-PARP-1 and cleaved-Caspase 3

Considering the close association of PGC-1α with anchorage-independent growth and invasion of cancer cells, we further investigated whether PGC-1α is involved in the anoikis-resistance of NPC cells induced by LMP1. First, we found that no significant difference in the PGC-1α transcriptional level between LMP1-negative and LMP1-positive NPC cells in suspension culture (Fig. 2A). However, higher expression of PGC-1α protein was observed in the latter group (Fig. 2B). Moreover, knockdown of LMP1 attenuated PGC-1α protein expression (Fig. 2B). These results suggest that LMP1 up-regulates the protein expression of PGC-1α in NPC cells. Next, we observed that PGC-1α re-expression in LMP1-negative cells cultured in suspension increased cell survival rate as well as colony formation; whereas PGC-1α silencing in LMP1-positive cells impeded both cell survival and the number of colonies (Fig. 2C-D). In addition, the detection of apoptotic markers, PARP-1 and cleaved-Caspase 3, further confirmed the above observations (Fig. 2E). Activation of the EMT and PI3-K/Akt signaling pathway is the most common mechanism endowing cancer cells with anoikis resistance [54–56]. We found that PGC-1α re-expression promoted EMT and activated the PI3-K/Akt pathway (Supplementary Fig. 3). Additionally, PTEN, the most important negative regulator of PI3-K/Akt signaling, was significantly inhibited by PGC-1α (Supplementary Fig. 3). Taken together, these results indicate that PGC-1α mediates the anoikis resistance of NPC cells induced by LMP1.

### LMP1 enhances the stability of PGC-1α by promoting the PRMT1-PGC-1α interaction to induce arginine methylation of PGC-1α

**Fig. 3:**
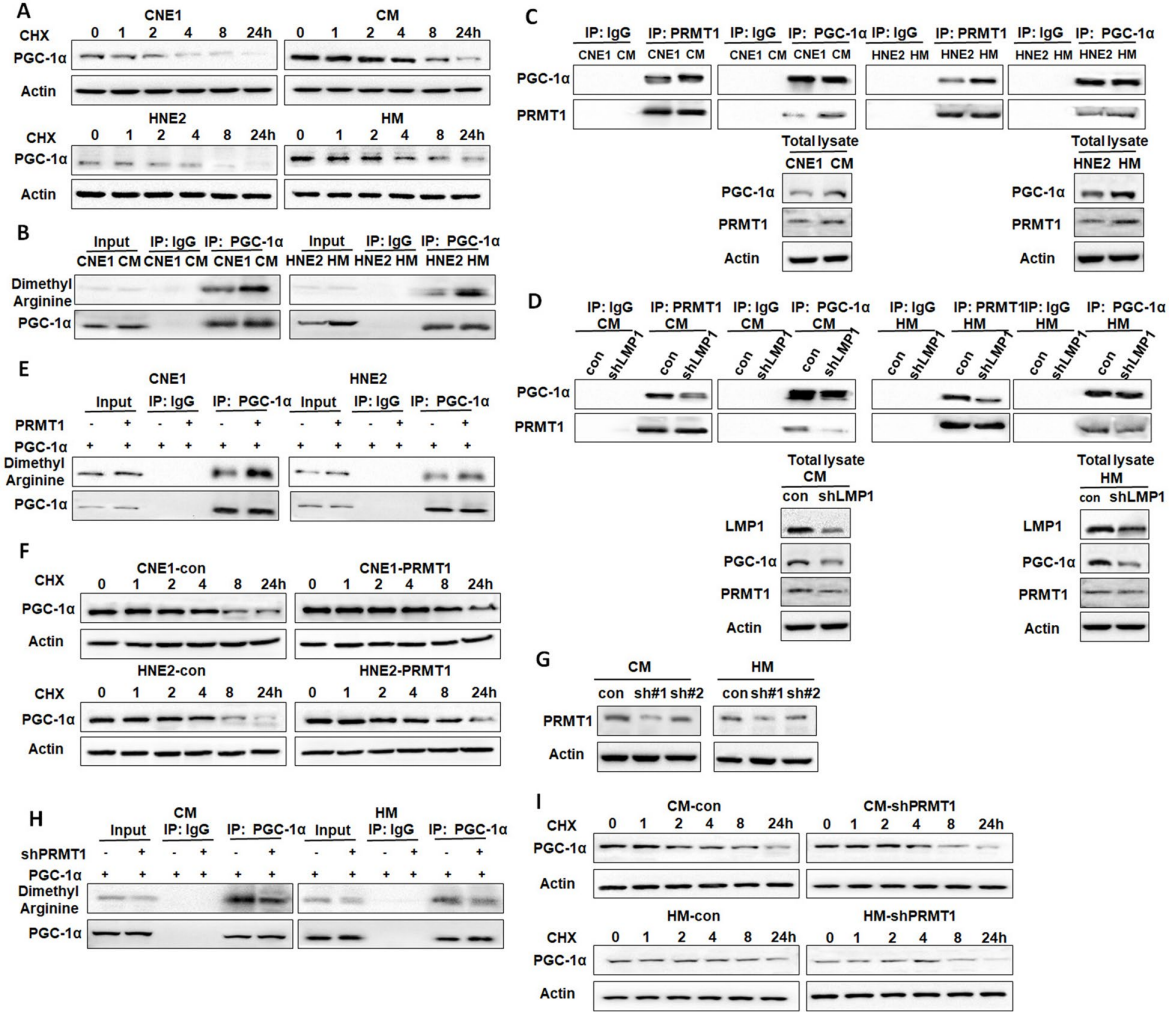
LMP1 promotes the PRMT1-PGC-1α interaction and induces arginine methylation of PGC-1α to enhance the stability of PGC-1α. **A** CNE1/CM and HNE2/HM cells were cultured in suspension and treated with CHX for 0, 1, 2, 4, 8, and 24 h, respectively, and the expression of PGC-1α protein was detected by Western blotting. **B** CNE1/CM and HNE2/HM cells were cultured in suspension for 48 h, and the arginine methylation level of PGC-1α was detected by Western blotting after purification of PGC-1α. **C** CNE1/CM and HEN2/HM cells were cultured in suspension, and the interaction between PRMT1-PGC-1α was detected by co- immunoprecipitation. **D** LMP1 was knocked down in CM and HM cells. Cells were cultured in suspension, and the interaction between PRMT1-PGC-1α was detected by co- immunoprecipitation. **E** PRMT1 was overexpressed in CNE1 and HNE2 cells, cultured in suspension for 48 h, and the arginine methylation level of PGC-1α was detected by Western blot analysis after purification of PGC-1α. **F** PRMT1 was overexpressed in CNE1 and HNE2 cells. The cells were cultured in suspension and treated with CHX for 0, 1, 2, 4, 8 and 24 h, respectively, and the expression of PGC-1α protein was detected by Western blotting. **G** The shRNA targeting PRMT1 was synthesized, and shRNA was transfected to CM and HM cells for 48 h, and then the expression level of PRMT1 was detected by Western blot analysis. **H** PRMT1 was knocked down in CM and HM cells. Cells were cultured in suspension for 48 h, and the arginine methylation level of PGC-1α was detected by Western blotting after purification of PGC-1α. **I** PRMT1 was knocked down in CM and HM cells. The cells were cultured in suspension and treated with CHX for 0, 1, 2, 4, 8 and 24 h, respectively, and the expression of PGC-1α protein was detected by Western blot analysis

PGC-1α is a short-lived protein whose cellular level is tightly controlled by a dynamic balance of synthesis and degradation. On the basis of our findings that LMP1 up-regulated the protein expression of PGC-1α, but not the mRNA level, we reasoned that LMP1 might regulate PGC-1α at the post-translational level. The results showed that the half-life of PGC-1α protein in LMP1-positive NPC cells was markedly prolonged compared to LMP1-negative NPC cells (Fig. 3A), suggesting that LMP1 enhances the stability of PGC-1α. Multiple post-translational modifications have an impact on PGC-1α. Among them, only Thr/Ser phosphorylation of PGC-1α has been reported to affect its stability. Thus, we determined the pan-Thr/Ser phosphorylation levels in LMP1-negative and LMP1-positive NPC cells, and found that no obvious phosphorylation was observed in either cell line (Supplementary Fig. 4). The data also indicated that phosphorylation modification might not contribute to the enhanced stability of PGC-1α induced by LMP1. Considering that arginine methylation is involved in a variety of intracellular regulatory processes, including protein stability, we further measured the arginine methylation level of PGC-1α in CNE1, CM, HNE2, and HM cells, respectively, and found that LMP1 promoted the arginine methylation of PGC-1α (Fig. 3B). As a major member of PRMT family, PRMT1 is responsible for transferring methyl groups from S-adenosylmethionine to the guanido nitrogens of arginine residues to form monomethyl and dimethylarginine [47, 48]. Arginine methylation by PRMT1 has been implicated in the regulation of signal transduction, transcriptional activation, and protein stability, and PGC-1α was identified as one of several arginine methylation substrates for PRMT1 [39, 57, 58]. In that PRMT1 is an interacting protein for LMP1 [59], we further examined whether LMP1 could recruit PRMT1 to up-regulate the arginine methylation level and the consequent protein stability of PGC-1α. Firstly, using a Co-IP experiment, we found that PRMT1 interacted with PGC-1α, and LMP1 promoted the binding of PRMT1 to PGC-1α (Fig. 3C). Knockdown of LMP1 in CM or HM cells attenuated the interaction between PRMT1 and PGC-1α (Fig. 3D). Secondly, we re-expressed PRMT1 in LMP1-negative cells and observed increased arginine methylation of PGC-1α (Fig. 3E). Moreover, PRMT1 re-expression extended the half-life of PGC-1α (Fig. 3F). Next, we designed short hairpin RNAs (shRNAs, sh#1 and sh#2) targeting PRMT1 and confirmed that sh#1 exhibited higher knockdown efficiency (Fig. 3G). Thus, sh#1 was used for the subsequent experiments. PRMT1 knockdown in LMP1-positive cells cultured with suspension decreased the arginine methylation level of PGC-1α (Fig. 3H); and the protein degradation rate of PGC-1α was accelerated (Fig. 3I). Additionally, we found that PRMT1 protein expression was not significantly affected by LMP1 (Supplementary Fig. 5). Therefore, these data indicate that LMP1 up-regulates the arginine methylation of PGC-1α by promoting the interaction between PRMT1 and PGC-1α, thus improving the protein stability of PGC-1α.

### The C-terminal CTAR-2 domain of LMP1 promotes PRMT1-PGC-1α interaction to enhance PGC-1α arginine methylation

**Fig. 4:**
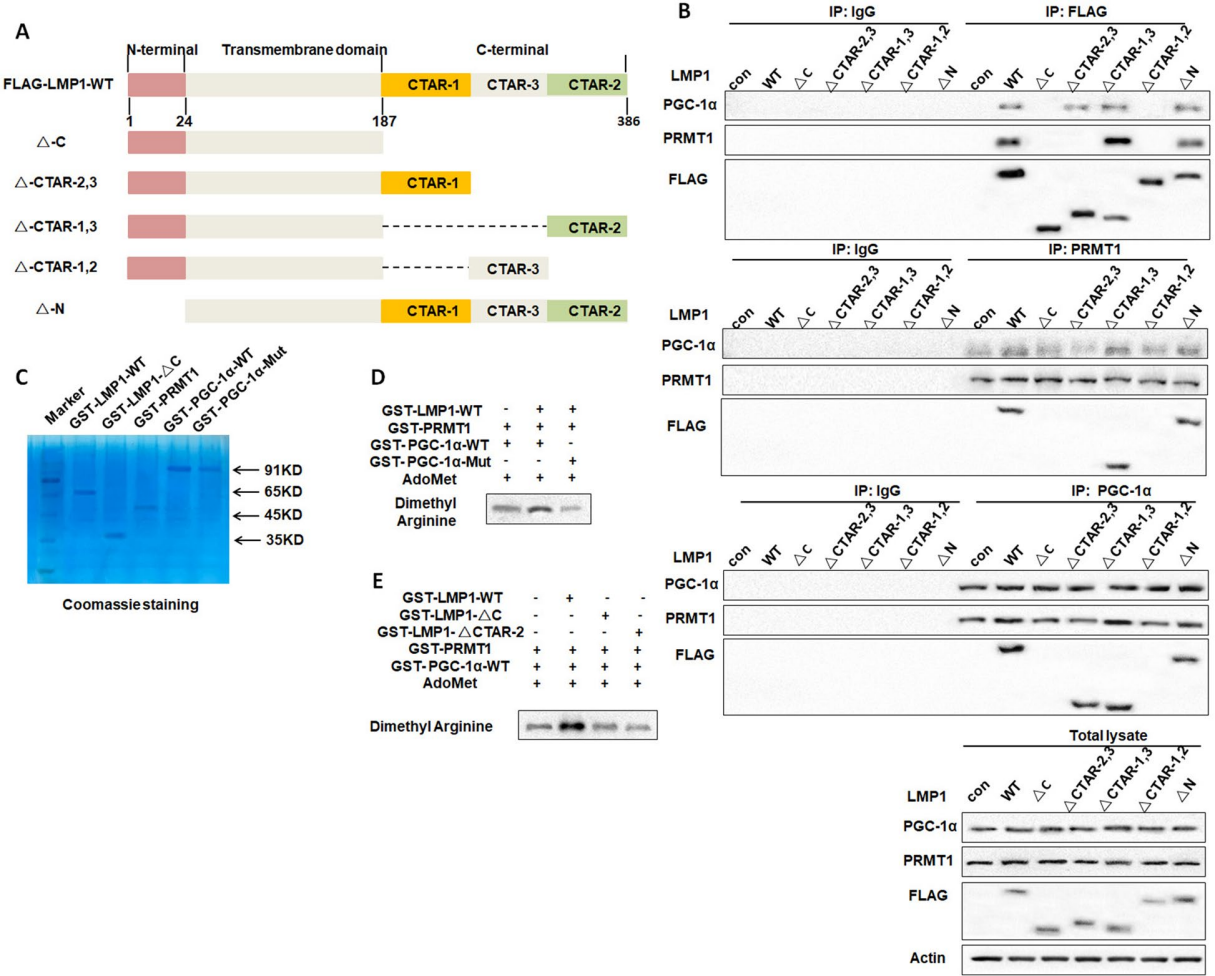
The C-terminal CTAR-2 domain of LMP1 promotes the interaction of PRMT1 with PGC-1α to enhance PGC-1α arginine methylation. **A** Structure diagram of each truncation of LMP1. **B** The LMP1 truncated plasmid, PRMT1 overexpression plasmid, and PGC-1α overexpression plasmid were simultaneously overexpressed in 293T cells, and the PRMT1-PGC-1α interaction was detected by co-immunoprecipitation. **C** GST-LMP1-WT, GST-LMP1-△C, GST-PRMT1, GST-PGC-1α-WT, and GST-PGC-1α-Mut fusion proteins were purified, and then visualized by Coomassie brilliant blue staining. **D** The GST-LMP1-WT and GST-PRMT1 proteins were incubated with GST-PGC-1α-WT or mutant protein GST-PGC-1α-Mut and AdoMet for in vitro methylation reaction. The level of arginine methylation modification of PGC-1α was detected by Western blotting. **E** GST-LMP1-WT, GST-LMP1-△C, and GST-LMP1-△CTAR-2 were incubated with GST-PRMT1 protein, GST-PGC-1α-WT protein, and AdoMet, respectively, and the level of arginine methylation modification of PGC-1α was detected by Western blot analysis

In order to identify the specific domain of LMP1 that promotes the PRMT1-PGC-1α interaction, we predicted the complex structure of LMP1, PRMT1, and PGC-1α by AlphaFold- Multimer using the supercomputing resource from Primary Biotech [60]. The sequences of CTAR-1 and CTAR-2 from LMP1 were submitted separately to AlphaFold-Multimer with the full sequence of PRMT1 and PGC-1α. In the complex structure of CTAR-1/PRMT1/PGC-1α, the CTAR-1 peptide bound to PGC-1α and was located far away from the interaction surface of PRMT1 (Supplementary Fig. 6A), forming interactions with only residues from PGC-1α. In the CTAR-2/PRMT1/PGC-1α complex structure, the CTAR-2 peptide bound upon the contact surface of PRMT1 and PGC-1α, forming interactions with both proteins (Supplementary Fig. 6B). Next, Co-IP experiments were performed to verify the complex structure prediction. HEK293T cells were co-transfected with plasmids expressing wild type or truncated flag-LMP1 (Fig. 4A), PRMT1, and PGC-1α. We found that C-terminal deletion of LMP1 resulted in attenuated PRMT1 interaction with PGC-1α. However, the deletion of the N-terminus of LMP1 did not affect the binding of PRMT1 to PGC-1α (Fig. 4B), indicating that the domain affecting the binding of PRMT1 to PGC-1α was located at the C-terminus of LMP1. Further results showed that the deletion of CTAR-2 domain at the C-terminus of LMP1 (△CTAR-2,3 or △CTAR-1,2) resulted in a sharp reduction of the PRMT1-PGC-1α interaction (Fig. 4B). These data suggest that LMP1 promotes the interaction between PRMT1 and PGC-1α through its C-terminal CTAR-2 domain.

Based on the observations that LMP1 enhanced PGC-1α arginine methylation, and CTAR-2 domain was essential for the promotion of the PRMT1-PGC-1α interaction induced by LMP1, we proposed that LMP1-CTAR-2 might mediate the up-regulation of PGC-1α methylation modification. Studies have shown that the arginine methylation modification of PGC-1α mainly occurs at arginine residues 665, 667, and 669. Thus, we constructed plasmids expressing GST fusion proteins of wild type PGC-1α (GST-PGC-1α-WT), mutated PGC-1α (GST-PGC-1α-Mut, with the mutation of arginine at 665, 667 and 669), wild type LMP1 (GST-LMP1-WT), truncated LMP1 (C-terminal deletion: GST-LMP1-△C, CTAR-2 domain deletion: GST-LMP1-△CTAR-2), and PRMT1 (GST-PRMT1), respectively. The fusion proteins were purified (Fig. 4C), and then used for an in vitro methylation assay. We demonstrated that PRMT1 can methylate PGC-1α and this action was enhanced by LMP1. When the methylation sites of PGC-1α (R665, R667 and R669) were mutated, the modification of PGC-1α was greatly attenuated (Fig. 4D). More importantly, C-terminal or CTAR-2 deletion remarkably reversed the arginine methylation modification of PGC-1α enhanced by LMP1 (Fig. 4E). Taken together, these results illustrate that the CTAR-2 domain of LMP1 induces the arginine methylation modification of PGC-1α by promoting the PRMT1-PGC-1α interaction, thereby enhancing the stability of PGC-1α.

### PRMT1 mediates LMP1-inhibited PGC-1α ubiquitination by enhancing arginine methylation of PGC-1α

**Fig. 5:**
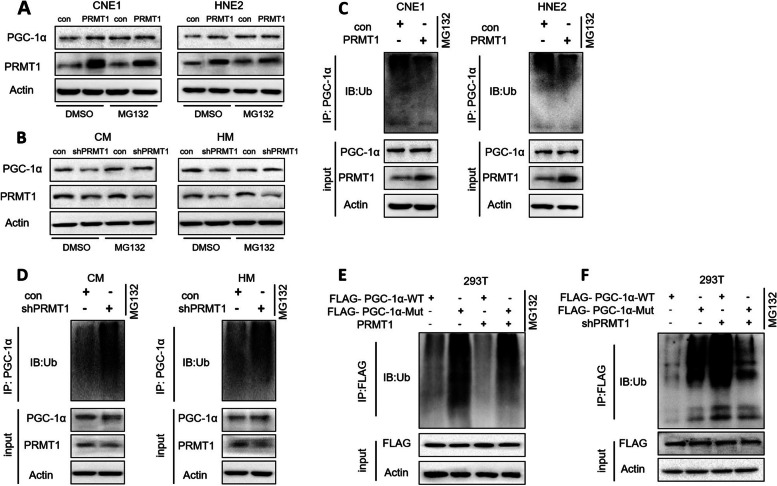
PRMT1 mediates LMP1-inhibited PGC-1α ubiquitination by enhancing arginine methylation of PGC-1α. **A** PRMT1 was overexpressed in CNE1 and HNE2 cells. The cells were cultured in suspension and treated with the proteasome inhibitor MG132, and the expression of PGC-1α was detected by Western blotting. **B** PRMT1 was knocked down in CM and HM cells. The cells were cultured in suspension and treated with the proteasome inhibitor MG132. The expression of PGC-1α was detected by Western blotting. **C** PRMT1 was overexpressed in CNE1 and HNE2 cells. The cells were cultured in suspension and treated with the proteasome inhibitor MG132, and the ubiquitination level of PGC-1α was detected by in vivo ubiquitination assay. **D** PRMT1 was knocked down in CM and HM cells. The cells were cultured in suspension and treated with the proteasome inhibitor MG132, and the ubiquitination level of PGC-1α was detected by in vivo ubiquitination assay. **E** The FLAG-PGC-1α-WT/FLAG-PGC-1α-Mut and PRMT1 plasmids were transfected into 293T cells, and then 293T cells were treated with proteasome inhibitor MG132. The ubiquitination level of PGC-1α was then detected by in vivo ubiquitination assay. **F** The FLAG-PGC-1α-WT/FLAG-PGC-1α-Mut and shPRMT1 plasmids were transfected into 293T cells. The 293T cells were treated with the proteasome inhibitor MG132, and the ubiquitination level of PGC-1α was detected by in vivo ubiquitination assay

Previous studies have shown that PGC-1α can be degraded through the proteasome pathway [61]. To explore whether PRMT1 mediates LMP1 affecting the stability of PGC-1α through the ubiquitin–proteasome pathway, we transfected a PRMT1-expression plasmid or the negative control into LMP1-negative cells, and treated them with the vehicle (DMSO) or MG132, a proteasomal inhibitor. The results showed that overexpression of PRMT1 up-regulated PGC-1α in the vehicle group, but not so convincingly in MG132-treated cells (Fig. 5A). Furthermore, knocking down PRMT1 in LMP1-positive cells also supported this conclusion (Fig. 5B). These data suggest that PRMT1 might promote the protein stability of PGC-1α by inhibiting the proteasome pathway. In addition, re-expression of PRMT1 in LMP1-negative cells reduced the ubiquitination level of PGC-1α (Fig. 5C). In contrast, knockdown of PRMT1 in LMP1-positive cells enhanced PGC-1α ubiquitination (Fig. 5D). We further mutated R665, R667, and R669 of PGC-1α (PGC-1α-Mut) to prevent PGC-1α from being methylated by PRMT1. In vivo ubiquitination experiments showed that overexpression of PRMT1 markedly reduced the ubiquitination level of PGC-1α-WT, whereas it had no significant effect on PGC-1α-Mut (Fig. 5E), suggesting that PRMT1 inhibits PGC-1α ubiquitination by up-regulating arginine methylation of PGC-1α at R665, R667, and R669. The results from knockdown of PRMT1 further support this conclusion (Fig. 5F). Together, these data indicate that PRMT1 mediates LMP1-inhibited PGC-1α ubiquitination by promoting arginine methylation of PGC-1α.

### PGC-1α mediates LMP1-promoted immune escape in NPC cells by upregulating PD-L1

**Fig. 6:**
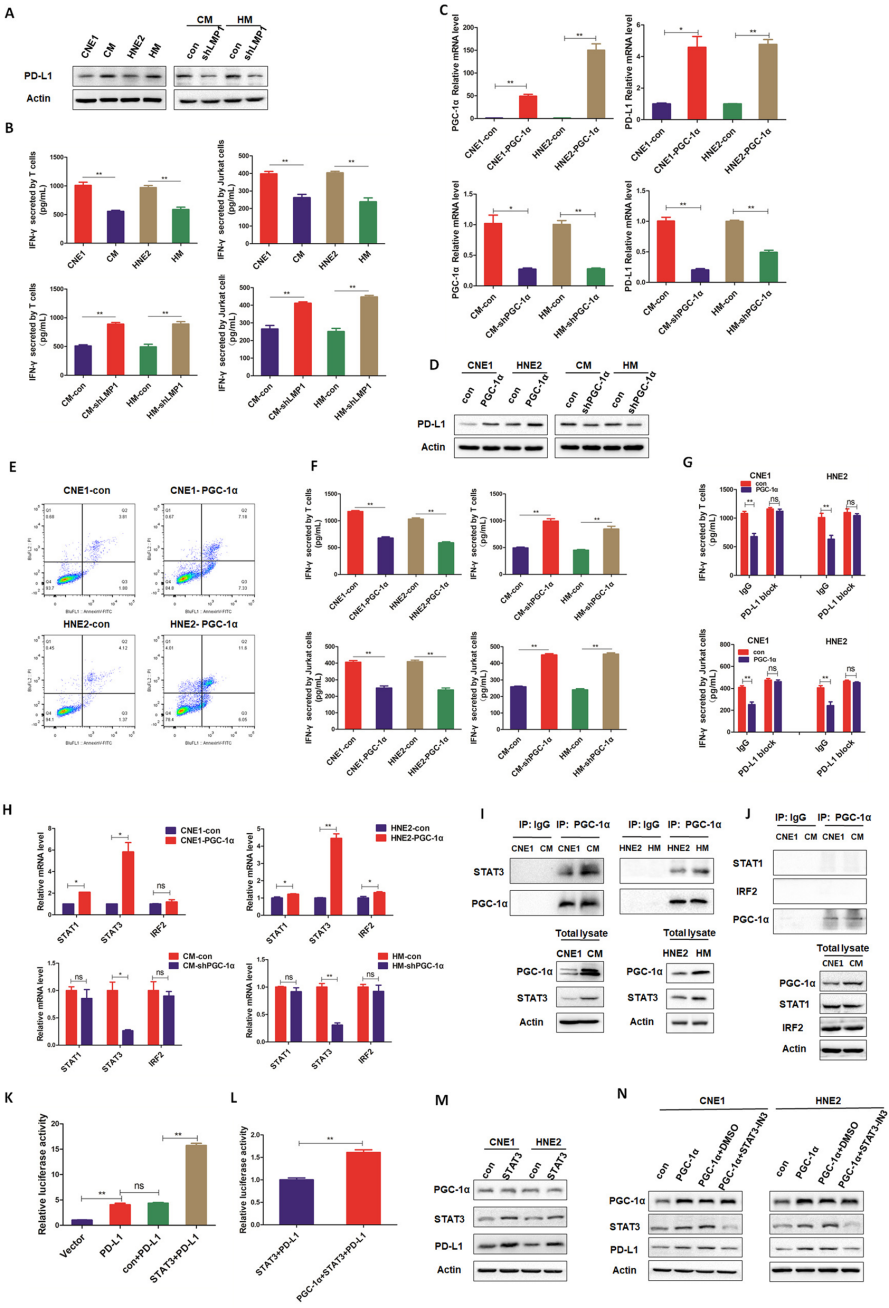
PGC-1α mediates LMP1 upregulation of PD-L1 to promote immune escape of NPC cells. **A** CNE1/CM, HNE2/HM, CM-con/CM-shLMP1, and HM-con/HM-shLMP1 cells were cultured in suspension for 48 h, and the protein levels of PD-L1, TNFα and VEGF were detected by Western blotting. **B** CNE1/CM, HNE2/HM, CM-con/CM-shLMP1, and HM-con/HM-shLMP1 cells were co-cultured with Jurkat cells or T cells for 24 h, and IFN-γ secreted by T cells was detected by ELISA (**p < 0.01, *p < 0.05). **C** PGC-1α was overexpressed in CNE1 and HNE2 cells or PGC-1α was knocked down in CM and HM cells, and the transcriptional levels of PGC-1α and PD-L1 were detected by real-time fluorescence quantitative PCR (**p < 0.01, *p < 0.05). **D** PGC-1α was overexpressed in CNE1 and HNE2 cells or PGC-1α was knocked down in CM and HM cells, and the protein expression level of PD-L1 was detected by Western blotting. **E** CNE1/HNE2 cells were transfected with PGC-1α overexpression plasmid and cultured for 48 h, and then co-cultured with T cells for 24 h, and the apoptotic rate of T cells was detected by flow cytometry. **F** PGC-1α was overexpressed in CNE1 and HNE2 cells or PGC-1α was knocked down in CM and HM cells. The secretion of IFN-γ by T cells was detected by ELISA (**p < 0.01, *p < 0.05). **G** PGC-1α was overexpressed in CNE1 and HNE2 cells, and then these cells were co-cultured with Jurkat cells or T cells. NPC cells were treated with sufficient anti-PD-L1 to block PD-L1 activity, and the secretion of IFN-γ in T cells was detected by ELISA (**p < 0.01, *p < 0.05). **H** PGC-1α was overexpressed in CNE1 and HNE2 cells or PGC-1α was knocked down in CM and HM cells, and the transcriptional levels of transcription factors STAT1, STAT3, and IRF2 were detected by real-time fluorescence quantitative PCR (**p < 0.01, *p < 0.05). **I** CNE1/CM and HNE2/HM cells were cultured in suspension, and the interaction of PGC-1α-STAT3 was detected by co-immunoprecipitation. **J** CNE1/CM cells were cultured in suspension, and the interaction of PGC-1α-STAT1 and PGC-1α-IRF2 was detected by co-immunoprecipitation. **K** Dual luciferase reporter assay was used to detect the effect of STAT3 on the PD-L1 promoter (**p < 0.01, *p < 0.05). **L** A dual luciferase reporter gene assay was used to detect the effect of PGC-1α and STAT3 on PD-L1 promoter (**p < 0.01, *p < 0.05). **M** CNE1/HNE2 cells were transfected with STAT3 overexpression plasmid, and the expression of PGC-1α, STAT3, and PD-L1 was detected by Western blotting. **N** CNE1/HNE2 cells were transfected with a PGC-1α overexpression plasmid and a STAT3 inhibitor was added; and the expression of PGC-1α, STAT3, and PD-L1 was detected by Western blotting

The activation of immunopathogenic PDL1/PD1 pathway between cancer cells and immune cells has been well described to inhibit antitumor immunity. We found that PD-L1 expression was up-regulated in LMP1-positive cells in comparison with that in LMP1-negative cells, and knockdown of LMP1 in CM and HM cells reduced the expression level of PD-L1 (Fig. 6A). In addition, the protein expression of other immune escape-related indicator TNF-α and VEGF was also examined, and similar results were observed (Fig. 6A). These results demonstrate that LMP1 enhances PD-L1 expression as well as other immune escape-related proteins, which suggests that LMP1 might affect immune tolerance of NPC cells. We further co-cultured CNE1/CM, HNE2/HM, CM-con/CM-shLMP1, and HM-con/HM-shLMP1 cells with Jurkat cells or T cells, and found that LMP1-positive cells significantly reduced IFN-γ secretion by T cell when compared to LMP1-negative cells (Fig. 6B). However, silencing LMP1 in LMP1-positive NPC cells increased IFN-γ secretion (Fig. 6B). These data indicate that LMP1 impedes the antitumor immunity of T cell and might facilitate the immune escape of NPC cells. Based on our previous findings showing that PGC-1α mediates LMP1-promoted anoikis resistance, we next determined whether PGC-1α is involved in LMP1-induced T cell dysfunction. PGC-1α was re-expressed in LMP1-negative cells or silenced in LMP1-positive cells. The data illustrated that both the mRNA and protein levels of PD-L1 are inducible by PGC-1α, which suggests that PGC-1α might act as an inducer of PD-L1 in NPC cells and mediates the up-regulation of PD-L1 by LMP1 (Fig. 6C-D). To access whether the up-regulation of PD-L1 by PGC-1α affects T cell function, we overexpressed PGC-1α in CNE1 and HNE2 cells or knocked down PGC-1α in CM and HM cells, and then co-cultured them with Jurkat cells or T cells. The results showed that PGC-1α re-expression in LMP1-negative NPC cells significantly induced T cell apoptosis (Fig. 6E) and inhibited IFN-γ secretion (Fig. 6F). In contrast, silencing PGC-1α in LMP1-positive NPC cells increased IFN-γ secretion (Fig. 6F). Notably, using anti-PD-L1 antibody to block PD-L1 expression, the inhibitory effect of PGC-1α on IFN-γ secretion by T cell was abolished (Fig. 6G). These data lend further support to the idea that PGC-1α mediates LMP1-promoted T cell dysfunction, which is dependent on the up-regulation of PD-L1.

As a typical co-activator, PGC-1α can interact with multiple transcription factors (TFs) to initiate the transcriptional program of target genes [62, 63]. To identify proximal mechanisms linked to the up-regulation of PD-L1 by PGC-1α, first, we screened out the potential TFs that might co-activate with PGC-1α to mediate LMP1-promoted PD-L1 expression. Signal transducers and activators of transcription 1, 3 (STAT1, STAT3) and interferon regulatory factor 2 (IRF2) have been reported to transcriptionally regulate PD-L1 expression [64–66]. The results manifested that among these transcriptional factors, STAT3 was the most inducible one by PGC-1α (Fig. 6H). More importantly, by using a Co-IP experiment, we found that PGC-1α interacted with STAT3, but not STAT1 or IRF2 (Fig. 6I-J). Moreover, stronger binding of PGC-1α to STAT3 was observed in LMP1-positive NPC cells compared to that in LMP1-negative cells. These data indicate that PGC-1α might interact and co-activate with STAT3, and LMP1 facilitates this interaction. Second, we performed transactivation assays to demonstrate that PGC-1α co-activates with STAT3 to boost PD-L1 transcription. We observed that STAT3 re-expression significantly enhanced the transcriptional activity of the *PD-L1* gene (Fig. 6K), and co-transfection of PGC-1α with STAT3 further enhanced the transcription of PD-L1 (Fig. 6L). Next, to interrogate whether the up-regulation of PD-L1 by PGC-1α is mediated by STAT3, we overexpressed STAT3 in LMP1-negative cells and found that PD-L1 protein expression was up-regulated, while it had no effect on PGC-1α protein expression (Fig. 6M). Moreover, PGC-1α was re-expressed in LMP1-negative cells with or without the addition of STAT3-IN3, a STAT3 inhibitor. The results showed that the up-regulation of PD-L1 by PGC-1α was attenuated by the STAT3 inhibition (Fig. 6N). In conclusion, these data indicate that PGC-1α mediates the up- regulation of PD-L1 by LMP1 by co-activating STAT3, which leads to T cell exhaustion and dysfunction, thus facilitating immune escape in NPC cells.

### PGC-1α mediates the invasion and metastasis of anoikis-resistant LMP1- positive NPC cells

**Fig. 7:**
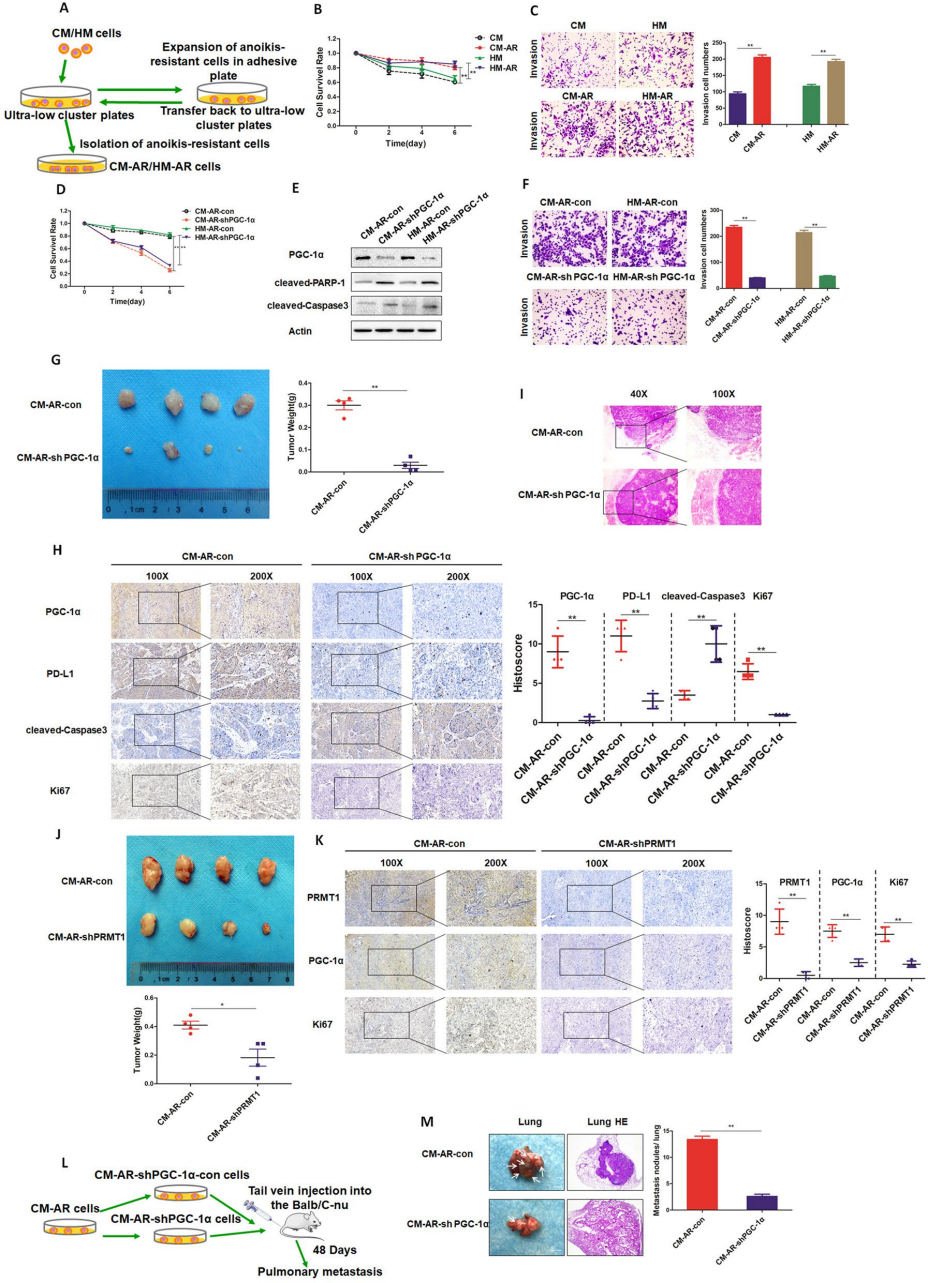
PGC-1α mediates the invasion and metastasis of anoikis-resistant LMP1-positive NPC cells. **A** Flow chart of anoikis-resistant LMP1-positive NPC cell lines. **B** The survival rates of CM/CM-AR and HM/ HM-AR cells were detected after 0, 2, 4, and 6 days of suspension culture (**p < 0.01, *p < 0.05). **C** CM/CM-AR and HM/HM-AR cells were cultured in suspension for 7 days, and the invasive ability of the cells was detected by cell invasion assay (**p < 0.01, *p < 0.05). **D** CM-AR-con/CM-AR-sh PGC-1α cells and HM-AR-con/HM-AR-sh PGC-1α cells were cultured in suspension, and the survival rate of cells was detected at 0, 2, 4, and 6 days, respectively (**p < 0.01, *p < 0.05). **E** CM-AR-con/CM-AR-shPGC-1α cells and HM-AR-con/HM-AR-shPGC-1α cells were cultured in suspension for 48 h, and the protein expression levels of cleaved-PARP-1 and cleaved-Caspase 3 were detected by Western blotting. **F** CM-AR-con/CM-AR-sh PGC-1α cells and HM-AR-con/HM-AR-sh PGC-1α cells were cultured in suspension for 7 days, and the invasive ability of the cells was detected by a cell invasion assay (**p < 0.01, *p < 0.05). **G** Nude mice were subcutaneously injected with CM-AR-con and CM-AR-shPGC-1α cells, and the tumor volume and mass were detected at 14 days (**p < 0.01, *p < 0.05). **H** Immunohistochemistry was used to analyze the expression of PGC-1α, PD-L1, cleaved-Caspase 3, and Ki67 in mouse tissue sections (**p < 0.01, *p < 0.05). **I** Nude mice were subcutaneously injected with CM-AR-con and CM-AR-shPGC-1α cells, and the infiltration of the tumor margins was detected by H&E staining. **J** Nude mice were subcutaneously injected with CM-AR-con and CM-AR-shPRMT1 cells, and the tumor volume and mass were detected at 14 days (**p < 0.01, *p < 0.05). **K** Immunohistochemistry was used to analyze the expression of PRMT1, PGC-1α, and Ki67 in mouse tissue sections (**p < 0.01, *p < 0.05). **L** Flow chart of lung metastasis of anoikis-resistant LMP1-positive NPC cells in nude mice. **M** CM-AR-con and CM-AR-shPGC-1α cells were injected into the tail vein of nude mice, and 48 days later, the lung metastatic nodules were observed by H&E staining (**p < 0.01, *p < 0.05)

To gain insight into the significance of PGC-1α inhibition in the invasion and metastasis of LMP1-positive NPC in vivo, first, we established anoikis-resistant cell lines from LMP1-positive CM and HM cells, named CM-AR and HM-AR, respectively (Fig. 7A). We examined the survival rate as well as invasion ability of CM-AR and HM-AR cells cultured in suspension, and observed that their survival rate was each significantly higher than that of CM and HM cells (Fig. 7B-C). Second, we stably transfected CM-AR and HM-AR cells with shPGC-1α, and found that the survival rates of CM-AR-shPGC-1α and HM-AR-shPGC-1α cells were markedly reduced compared to those of the control groups (Fig. 7D). Also, PGC-1α silencing increased the expression levels of apoptotic markers, cleaved-PARP-1 and cleaved-Caspase 3 (Fig. 7E). Moreover, PGC-1α knockdown led to attenuated invasive abilities of CM-AR and HM-AR cells (Fig. 7F). These results suggest that PGC-1α inhibition hampers the anti-anoikis and invasive capability of anoikis-resistant LMP1-positive NPC cells. Third, we performed xenograft experiments using CM-AR-shPGC-1α and the control CM-AR-con cells. PGC-1*α* silencing resulted in a remarkable suppression of tumor formation with approximate tenfold difference compared to the control CM-AR-con xenograft (Fig. 7G). Ki67 staining of the tumor xenograft tissues was also in consistent with this observation (Fig. 7H). We found that PGC-1α and PD-L1 staining was significantly decreased, while cleave-Caspase 3 was enhanced in the CM-AR-shPGC-1α group compared to the control (Fig. 7H). Infiltrative growth is a characteristic of invasive tumors. More importantly, we found that in the CM-AR- shPGC-1α group, the tumor had an intact capsule and clear boundary; whereas in CM-AR-con group, the capsule was discontinuous and the xenograft tumor had different degrees of infiltration with surrounding tissues (Fig. 7I). These results indicate that silencing of PGC-1α effectively impedes the growth and invasion of anoikis-resistant LMP1-positive NPC. Fourth, we established stable PRMT1 knockdown and the control cell lines from anoikis-resistant LMP1-positive CM, and used them for xenograft experiments. The results showed that the tumor size and mass of the shPRMT1 group were significantly reduced compared to those of the control group (Fig. 7J). IHC staining of the tumor xenograft tissue further demonstrated that silencing of PRMT1 to interfere with the interaction between PRMT1 and PGC-1α resulted in downregulated PGC-1α expression and the consequent growth inhibition of anoikis-resistant LMP1-positive NPC in vivo (Fig. 7K).

Next, we determined whether PGC-1α inhibition affects the metastatic capability of anoikis-resistant LMP1-positive NPC cells. CM-AR-shPGC-1α and CM-AR-con cells were injected into the tail veins of BALB/c nude mice, respectively. Mice were euthanized 48 days later, and lung tissues were isolated and fixed (Fig. 7L). The results illustrated that PGC-1α silencing led to a significant decrease of metastatic lung nodules when compared to the control group (Fig. 7M). H&E staining also confirmed this conclusion (Fig. 7M). In addition, the body weight of nude mice in the control group was significantly reduced in comparison with that in the shPGC-1α group (Supplementary Fig. 7). In addition, the GEPIA database analysis also indicated that PGC-1α expression was negatively correlated with the overall survival of patients with head and neck tumors (Supplementary Fig. 8).

## Discussion

EBV is well known to be the most common and persistent viral infection in humans. The complex interplay between EBV and its host largely contributes to a variety of malignancies, including NPC [1]. EBV-encoded proteins can utilize the PTM machinery of host cells by initiating specific protein–protein interactions, thus activating oncogenic signaling to facilitate the malignant phenotype of host cells [7].

**Fig. 8:**
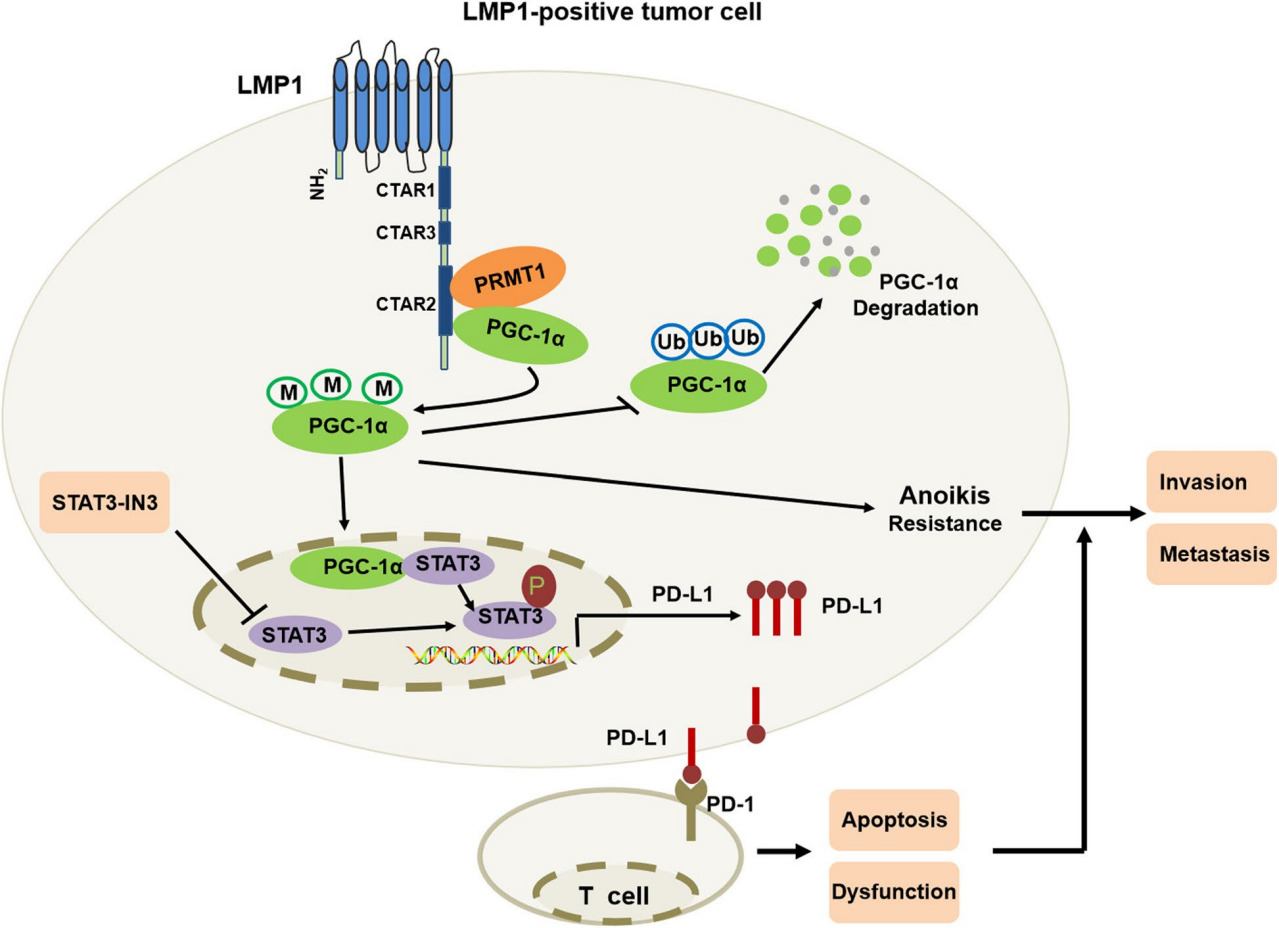
Mechanism of LMP1-induced stabilization of PGC-1α promoting invasion and metastasis of nasopharyngeal carcinoma. LMP1 promotes PRMT1-PGC-1α interaction to up-regulate the arginine methylation levels of PGC-1α. This action inhibits ubiquitination and degradation of PGC-1α, thus enhancing its protein stability. Elevated PGC-1α by LMP1 co-activates STAT3 to boost PD-L1 expression, consequently facilitating immune escape of cancer cells. These mechanisms coordinate to enhance the anoikis-resistance, invasion, and metastasis of cancer cells

As a major tumorigenic protein encoded by EBV, LMP1 extensively participates in oncogenic signal transduction through its functional domains. The N-terminal of LMP1 mediates cell proliferation; and the transmembrane region can polymerize and anchor LMP1 to membrane microdomain lipid rafts, leading to the activation of NF-κB, Rho-GTPases, and Cdc42 signaling. The CTAR1 and CTAR2 subdomains in the C-terminus are responsible for most of LMP1-mediated signal transduction [10]. In this work, we have demonstrated that LMP1 recruits and promotes the interaction between PRMT1 and PGC-1α, resulting in elevated arginine methylation levels and consequently enhancing the stability of PGC-1α (Fig. 8). By protein complex prediction using AlphaFold-Multimer algorithms, we found that the CTAR2 domain of LMP1 can bind with both PGC-1α and PRMT1. Co-IP and in vitro methylation assays confirmed that LMP1-CART2 is critical for the interaction between PGC-1α and PRMT1, and the consequent enhanced methylation of PGC-1α. The R665A, R667A, R669A mutants led to reduced methylation levels of PGC-1α, which suggests that the three amino acids might be crucial for the methylation modification of PGC-1α by PRMT1. Moreover, for the first time, we illustrated that PRMT1 mediates the LMP1-promoted methylation modification of PGC-1α, which protected it from degradation through the proteasome pathway and thus enhanced its stability. Accordingly, arginine methylation might be a novel post-translational modification for the regulation of PGC-1α stability.

Anoikis is considered to be a physiological barrier to metastasis; and avoiding anoikis is a major hallmark of metastasis [18, 54]. Biochemical studies have shown that multiple mechanisms contribute to enhanced cell anoikis-resistance, including the activation of the NF- κB/Src/Akt/ERK pathways, enzymes in integrin and growth factor receptors signaling, oxidative stress, EMT, and metabolic reprogramming [54, 55, 67–73]. These events trigger survival signals, inhibit apoptosis, counteract anoikis, and promote metastasis of cancer cells. In the present study, we demonstrate that PGC-1α mediates LMP1-enhanced anoikis-resistance, invasion, and metastasis of NPC cells. Overexpression of PGC-1α activated the PI3-K/Akt pathway and promoted EMT; however, PGC-1α knockdown attenuated the anoikis-resistant and invasive capability of LMP1-positive NPC cells. Moreover, we established anoikis-resistant LMP1-positive NPC cell lines to perform tumor xenograft and metastatic experiments in vivo. Our findings indicate that silencing of PGC-1α or knocking down of PRMT1 to interfere with its interaction with PGC-1α remarkably hampers the growth, invasion, and lung metastasis of anoikis-resistant LMP1-positive NPC cells.

Immune escape is a prerequisite for the survival and metastasis of cancer cells. The PD-1/PD-L1 signaling pathway represents the most studied immune checkpoint for cancer immunotherapy, and restoring T cell-mediated adaptive immunity has become one of the most frequently used strategies [51, 74]. In this study, we illustrated that PGC-1α mediates LMP1-induced immune tolerance of NPC cells. PGC-1α acts as a typical co-activator that interacts with multiple transcription factors, including PPARγ, nuclear respiratory factors (NRFs), and forkhead box class-O1 (FOXO1), to trigger rate-limiting steps in transcriptional initiation. We first demonstrated that PGC-1α can co-activate STAT3, but not STAT1 or IRF2, to boost PD-L1 expression, consequently resulting in the dysfunction of T lymphocytes. Up-regulation of PGC-1α markedly induced T cell apoptosis and hampered IFN-γ production, thus avoiding immune destruction of LMP1-positive NPC cells.

## Conclusion

Our work provides insights into how virus-encoded proteins recruit and interact with host regulatory elements to facilitate the malignant progression of NPC. On the one hand, LMP1 recruits and promotes the PRMT1-PGC-1α interaction to up-regulate the arginine methylation level of PGC-1α, thus enhancing its protein stability. Up-regulation of PGC-1α activates the PI3-K/Akt pathway and EMT to promote the anoikis-resistance and invasion of cancer cells. On the other hand, elevated PGC-1α by LMP1 co-activates with STAT3 to boost PD-L1 expression, consequently facilitating immune escape of cancer cells. The two mechanisms coordinate to enhance the anoikis-resistance, invasion, and metastasis of cancer cells. In addition, our findings also shed a light on the regulatory mechanism and function of post-translational modification of PGC-1α. Therefore, targeting PGC-1α or PRMT1-PGC-1α interaction might be exploited for therapeutic gain for EBV-associated malignancies.

### Supplementary Information

**Additional file 1: Supplementary figure 1.** (A) Cell viability of CNE1/CM, HNE2/HM, CM-con/ CM-shLMP1 and HM-con/HM-shLMP1 cells after 0, 12, 24 and 48h suspension. (B) Cell viability of C666-1-con/C666-1-shLMP1 cells after 0, 12, 24 and 48h suspension.

**Additional file 2: Supplementary figure 2.** (A) HNE2/HM and HM-con/HM-shLMP1 cells were cultured in suspension for 48 h, and the expression of Bcl-2 was detected by Western blotting. (B) C666-1-con/C666-1-shLMP1 cells were cultured in suspension for 48 h, and the expression of apoptotic marker cleaved-Caspase 3 and anoikis resistance-related proteins TrkB and Bcl-2 were detected by Western blotting.

**Additional file 3: Supplementary figure 3.** PGC-1α was overexpressed in CNE1 or HNE2 cells, culture cells in suspension, and the expression levels of PGC-1α, N-cadherin, Vimentin, E-cadherin, p-Akt, Akt and PTEN were detected by western blot.

**Additional file 4: Supplementary figure 4.** Pan phospho-serine/threonine level of PGC-1α in CNE1/CM, HNE2/HM cells was detected by western blot.

**Additional file 5: Supplementary figure 5.** CNE1/CM and HNE2/HM cells were cultured in suspension, and the expression level of PRMT1 was detected by western blot.

**Additional file 6: Supplementary figure 6.** The predicted complex structure of (A) CTAR-1/PRMT1/ PGC-1α and (B) CTAR2/PRMT1/PGC-1α. The proteins are showed in carton model. PRMT1 and PGC-1α are colored in cyan and green in both pictures; and CTAR-1 and CTAR-2 peptides are colored in orange and red, respectively. In the model of CTAR-1/PRMT1/PGC-1α, the peptide of CTAR-1 bound with PGC-1α and formed considerable interactions with I196, P202, H607, E648, K651 and Y654. In the model of CTAR-2/PRMT1/PGC-1α, the peptide of CTAR-2 contacts with both PRMT1 and PGC-1α, making interactions with F60, E64 and Y211 from PRMT1, and S266, P267, F275, K303, K372, K374, R375, F474, K694, E702 from PGC-1α.

**Additional file 7: Supplementary figure 7.** CM-AR-con and CM-AR-sh PGC-1α cells were injected into the tail veins of BALB/c nude mice. The body weight of the nude mice in each group was monitored.

**Additional file 8: Supplementary figure 8.** Survival analysis from TCGA dataset assessed by the Kaplan–Meier method for head and neck tumor patients with high or low signature of *PPARGC1A* genes.

**Additional file 9. **Supplemental Materials and Methods.

## Data Availability

Data sharing not applicable to this article as no datasets were generated or analyzed during the current study.

## Abbreviations

CM: CNE1-LMP1
Co-IP: Co-immunoprecipitation
CTAR1,2,3: C-terminal activating regions 1, 2, and 3
CTC: Circulating cancer cell
EBV: Epstein-Barr virus
EMT: Epithelial-mesenchymal transition
FBS: Fetal bovine serum
FOXO1: Forkhead box class-O1
HM: HNE2-LMP1
MEF: Mouse embryo fibroblasts
GC: Gastric carcinoma
IRF2: Interferon regulatory factor 2
LMP1: Latent membrane protein 1
MTS: 3-(4,5- Dimethylthiazol-2-yl)- 5-(3-carboxymethoxyphenyl)-2-(4- sulfophenyl)-2H- tetrazolium
NPC: Nasopharyngeal carcinoma
NRF: Nuclear respiratory factors
PD-1: Programmed cell death protein 1
PD-L1: Programmed cell death ligand 1
PGC-1α: Peroxisome proliferator activated receptor coactivator-1a
PPAR: Peroxisome proliferator activated receptor
PRMT1: Arginine methyltransferase 1
RIPK: Receptor interacting protein kinases
STAT: Signal transducers and activators of transcription
TF: Transcription factors
TM: Transmembrane domain
