# Supplementary figures and images for: Anoikis resistance and immune escape mediated by Epstein-Barr virus-encoded latent membrane protein 1-induced stabilization of PGC-1α promotes invasion and metastasis of nasopharyngeal carcinoma
Source: J Exp Clin Cancer Res. 2023 Oct 7;42:261. doi: 10.1186/s13046-023-02835-6 (PMC10559433; doi:10.1186/s13046-023-02835-6)

A

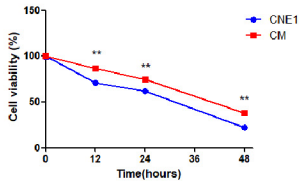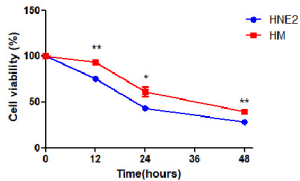

B

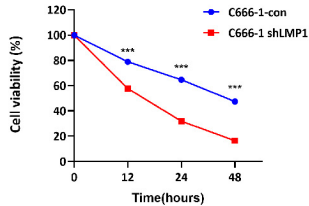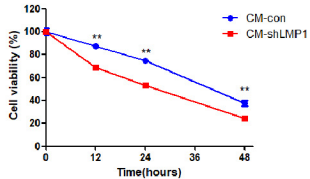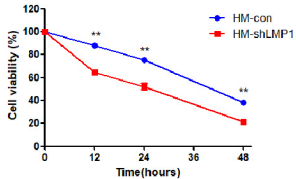

Supplement: Supplementary file 1 — Additional file 1: Supplementary figure 1. (A) Cell viability of CNE1/CM, HNE2/HM, CM-con/ CM-shLMP1 and HM-con/HM-shLMP1 cells after 0, 12, 24 and 48h suspension. (B) Cell viability of C666-1-con/C666-1-shLMP1 cells after 0, 12, 24 and 48h suspension. [file 13046_2023_2835_MOESM1_ESM.pdf]

**A**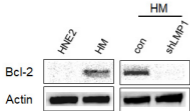**B**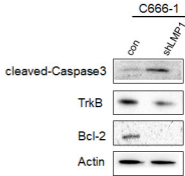

Supplement: Supplementary file 2 — Additional file 2: Supplementary figure 2. (A) HNE2/HM and HM-con/HM-shLMP1 cells were cultured in suspension for 48 h, and the expression of Bcl-2 was detected by Western blotting. (B) C666-1-con/C666-1-shLMP1 cells were cultured in suspension for 48 h, and the expression of apoptotic marker cleaved-Caspase 3 and anoikis resistance-related proteins TrkB and Bcl-2 were detected by Western blotting. [file 13046_2023_2835_MOESM2_ESM.pdf]

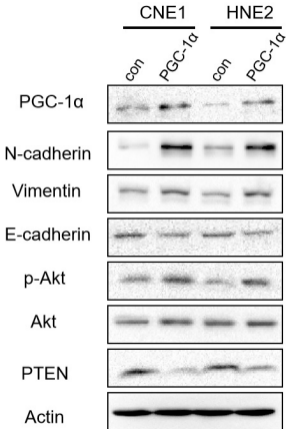

Supplement: Supplementary file 3 — Additional file 3: Supplementary figure 3. PGC-1α was overexpressed in CNE1 or HNE2 cells, culture cells in suspension, and the expression levels of PGC-1α, N-cadherin, Vimentin, E-cadherin, p-Akt, Akt and PTEN were detected by western blot. [file 13046_2023_2835_MOESM3_ESM.pdf]

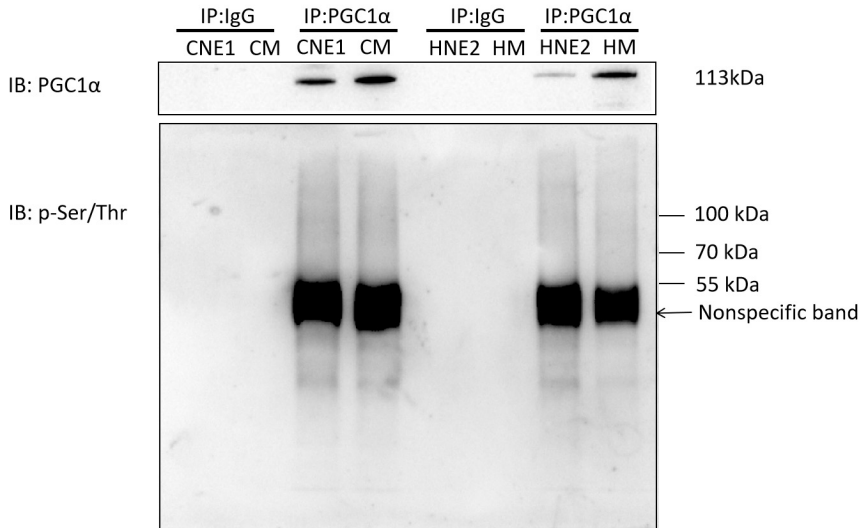

Supplement: Supplementary file 4 — Additional file 4: Supplementary figure 4. Pan phospho-serine/threonine level of PGC-1α in CNE1/CM, HNE2/HM cells was detected by western blot. [file 13046_2023_2835_MOESM4_ESM.pdf]

CNE1 CM HNE2 HM

PRMT1

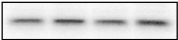

Actin

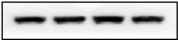

Supplement: Supplementary file 5 — Additional file 5: Supplementary figure 5. CNE1/CM and HNE2/HM cells were cultured in suspension, and the expression level of PRMT1 was detected by western blot. [file 13046_2023_2835_MOESM5_ESM.pdf]

**A**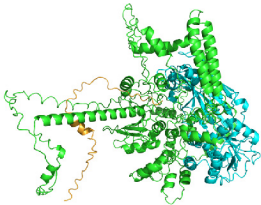**B**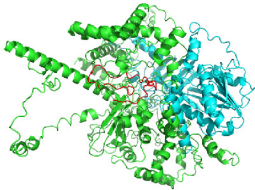

Supplement: Supplementary file 6 — Additional file 6: Supplementary figure 6. The predicted complex structure of (A) CTAR-1/PRMT1/ PGC-1α and (B) CTAR2/PRMT1/PGC-1α. The proteins are showed in carton model. PRMT1 and PGC-1α are colored in cyan and green in both pictures; and CTAR-1 and CTAR-2 peptides are colored in orange and red, respectively. In the model of CTAR-1/PRMT1/PGC-1α, the peptide of CTAR-1 bound with PGC-1α and formed considerable interactions with I196, P202, H607, E648, K651 and Y654. In the model of CTAR-2/PRMT1/PGC-1α, the peptide of CTAR-2 contacts with both PRMT1 and PGC-1α, making interactions with F60, E64 and Y211 from PRMT1, and S266, P267, F275, K303, K372, K374, R375, F474, K694, E702 from PGC-1α. [file 13046_2023_2835_MOESM6_ESM.pdf]

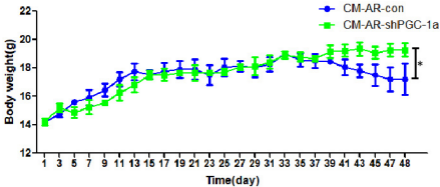

Supplement: Supplementary file 7 — Additional file 7: Supplementary figure 7. CM-AR-con and CM-AR-sh PGC-1α cells were injected into the tail veins of BALB/c nude mice. The body weight of the nude mice in each group was monitored. [file 13046_2023_2835_MOESM7_ESM.pdf]

# Overall Survival

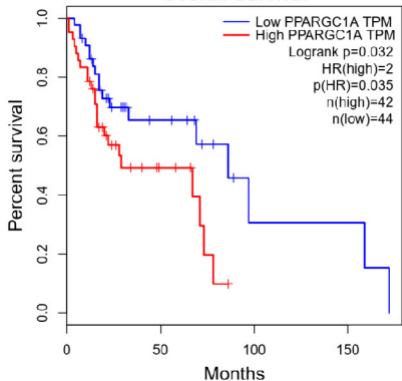

Supplement: Supplementary file 8 — Additional file 8: Supplementary figure 8. Survival analysis from TCGA dataset assessed by the Kaplan–Meier method for head and neck tumor patients with high or low signature of PPARGC1A genes. [file 13046_2023_2835_MOESM8_ESM.pdf]
