# Supplementary material for: Anoikis resistance and immune escape mediated by Epstein-Barr virus-encoded latent membrane protein 1-induced stabilization of PGC-1α promotes invasion and metastasis of nasopharyngeal carcinoma
Source: J Exp Clin Cancer Res. 2023 Oct 7;42:261. doi: 10.1186/s13046-023-02835-6 (PMC10559433; doi:10.1186/s13046-023-02835-6)
Supplement: Supplementary file 9 — Additional file 9. Supplemental Materials and Methods. [file 13046_2023_2835_MOESM9_ESM.docx]

**Supplemental Materials and Methods**

**Cell viability assay**

The cell proliferation assay was performed using Cell Counting Kit-8 (C0038, Beyotime). Cells were cultured in a 96-well plate for 0, 12, 24, and 48 h, respectively, and then the assay solution was added and incubated for 2 h at 37℃ in the dark. A microplate reader (Beckman, Brea, CA, USA) was used to measure the results at 490 nm.

**Clonogenic survival assay**

After suspension culture of CNE1, CNE1-LMP1, HNE2, and HNE2-LMP1 cells in ultra-low-attachment 6-well plates (3471, Corning), the cells were incubated in 24-well plates until cell clones were formed. The cells were then stained with 0.5% crystal violet and counted.

**Cell invasion assay**

The cell invasion assay was conducted in a 24-well matrix gel invasion chamber. The upper surface of the invasion chamber was covered with matrix gel (356234, Corning), and the filter hole was 8.0 μm. Briefly, the upper chamber was inoculated with 2 × 10^5^ cells in serum-free medium, and the lower chamber was supplemented with 10% FBS medium. After a 72h incubation, the cells were stained with 0.5% crystal violet solution. The number of invading cells was randomly counted under a microscope.

**Western blot and co-immunoprecipitation (Co-IP) analysis**

Cells were disrupted with IP lysis buffer (PC104, EpiZyme) to obtain total proteins. Protein concentration was determined using a BCA assay reagent (Pierce Chemical, Rockford, IL, USA) in accordance with the manufacturer's protocol. For immunoblotting, cell lysates were separated by SDS-PAGE, blotted with appropriate primary antibody followed by an HRP-labeled secondary antibody, and then detected using a chemiluminescence solution (Us Everbright, San Ramon, CA, USA). The ChemiDoc XRS system and Image Lab software (Bio-Rad, Hercules, CA, USA) were used for protein visualization. For immunoprecipitation, cell lysates were separated and clarified by immunomagnetic beads and incubated overnight with indicator antibodies plus Dynabeads®Protein A/G (B23202, Bimake) at 4℃ to form an immune complex. After extensive washing with PBS, the immune complexes were analyzed by Western blotting as described above.

**Protein half-life assays**

The cells were treated with cycloheximide (20 μg/mL, Saint Louis, MO, USA) for different periods to block protein synthesis. Western blot analysis was used to detect protein levels.

**Immunohistochemistry (IHC)**

The tissue samples from NPC patients were collected from the Xiangya Hospital of Central South University. IHC was performed using a SP Broad Spectrum kit (SP0041, Solarbio). After treatment with 3% H_2_O_2_, the tissue sections were blocked with 5% normal goat serum and incubated overnight with appropriate primary antibody at 4°C. Then, the sections were sequentially incubated with biotin anti-mouse/rabbit IgG for 30 min followed by streptomyces anti-biotin-peroxidase for 30 min and then stained with DAB for 5-30 min. Stained slides were scored according to the intensity of staining (0: -, 1: +, 2: ++, and 3: +++) and the percentage of the cells of interest staining positive for each antigen (0: 0%, 1: 1–25%, 2: 26–50%, 3: 51–75% and 4: 76–100%). The intensity score was multiplied by the percentage score to obtain the final score for statistical analysis.
